# Additions to the myxobiota of the Åland Islands

**DOI:** 10.3897/BDJ.3.e4653

**Published:** 2015-03-09

**Authors:** Panu Kunttu, Elina Varis, Sanna-Mari Kunttu

**Affiliations:** ‡ University of Eastern Finland, Joensuu, Finland; § Virontörmänkatu 1 D 18, Tampere, Finland; | Metsähallitus, Parks & Wildlife Finland, Dalsbruk, Finland

**Keywords:** Amoebozoa, Biogeography, Myxogastria, Myxomycetes, Slime mold

## Abstract

Six myxomycete species new to the Åland Islands are presented: *Comatricha elegans*, *Cribraria intricata*, *Didymium minus*, *Hemitrichia clavata*, *Licea variabilis* and *Trichia favoginea*. The record of *Cribraria intricata* is the third in Finland. Specimens were collected in September 2014. Altogether the number of myxomycete species found from the Åland Islands is now 55.

## Introduction

Myxomycetes (or Myxogastria) - commonly known as slime molds - form a group of fungus-like eukaryotic microorganisms. However, in systematics they belong to the division of Amoebozoa or they are classified as amoeboid protists (Fiore-Donno et al. 2010, Stephenson 2011). Their status in systematics has been unclear and unstable, and partly it is that still.

The myxomycetes constitute a relatively small group around 1000 species worldwide (Lado 2014). Most of them are cosmopolitans and endemic myxomycete species are not known (Martin and Alexopoulos 1969). Nevertheless, some species appear to have geographically restricted distribution (Ing 1994, Estrada-Torres et al. 2013). Myxomycetes occur mostly in all kind of terrestrial environments, but few aquatic species also exist (Lindley et al. 2007). Due to a limited number of active researchers, the knowledge of distribution and ecology is largely reflected with records from regions where most of the myxomycetologists live and work.

Altogether 215 myxomycete species are found in Finland (Härkönen and Varis 2012, Kunttu et al. 2013, Pennanen 2014). The knowledge of occurrence is limited in the Åland Islands: before this study 49 species were found from the Åland Islands (Härkönen and Varis 2012, Kunttu et al. 2013, Kunttu et al. 2014).

**Figure 1.**
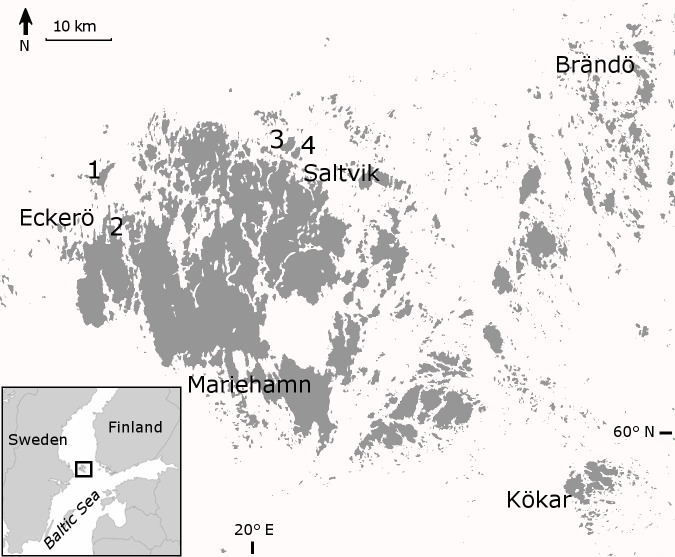
Location of Åland Islands and the study sites: 1) Finbo, 2) Svartnö, 3) Boxö and 4) Boxö ön.

The Åland Islands are a large archipelago area between Finland and Sweden in Fennoscandia. The archipelago is situated in the middle of the Baltic Sea (Fig. 1). These islands constitute their own biogeographic province called Alandia and it is located in the hemiboreal zone (Knudsen and Vesterholt 2012). Land area is approximately 1552 km^2^ and it comprises of 6757 islands and islets, each of an area at least 0.25 hectares (Åkerberg 2010). The nature of Åland Islands comprises many special features in a Finnish scale, like long growing season (195 days), broad diversity of habitats, commonly occurring herb-rich forests, semi-natural woodlands and meadows which are still commonly grazed by domestic animals. The volume of dead wood is the highest in Finland (Juntunen 2014).

## Materials and methods

**Study sites**

**Figure 2.**
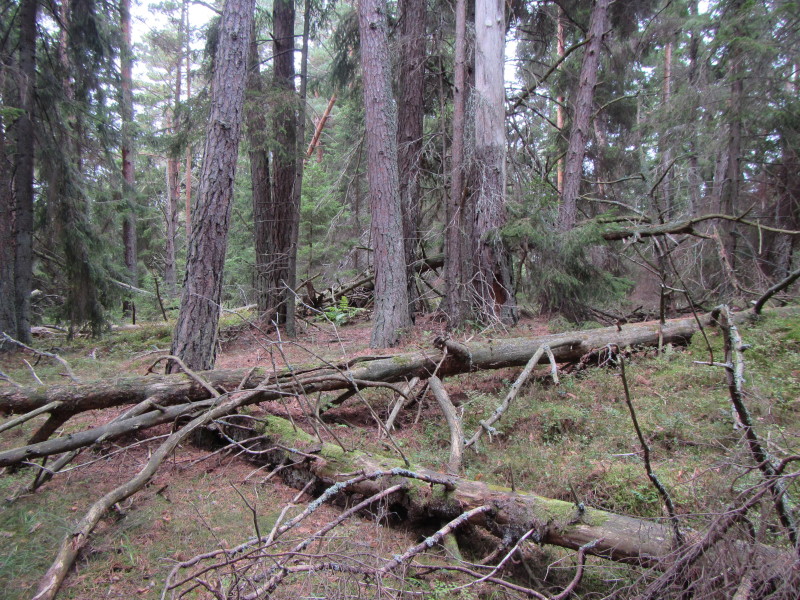
Habitat of *Cribraria intricata* on the island of Svartnö, in Eckerö municipality.

**Figure 3.**
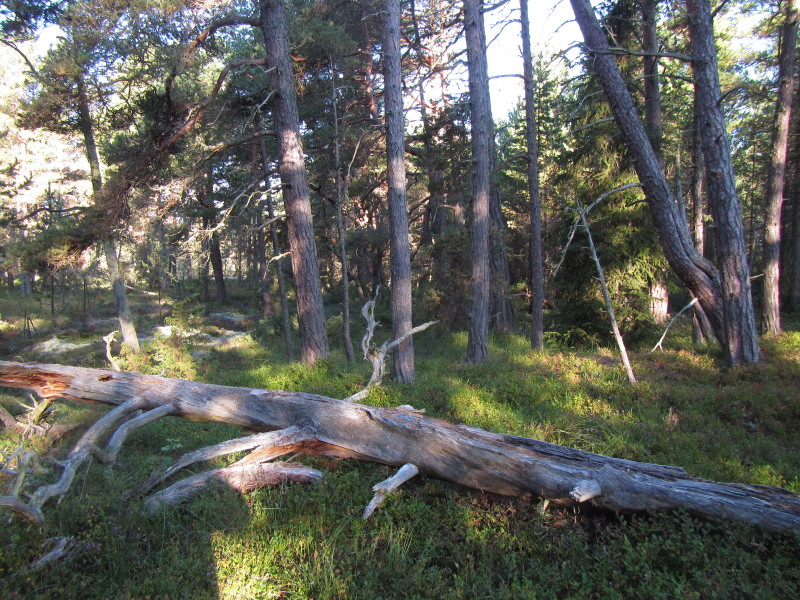
Habitat of *Comatricha elegans* and *Licea variabilis* on the island of Finbo, in Eckerö municipality.

**Figure 4.**
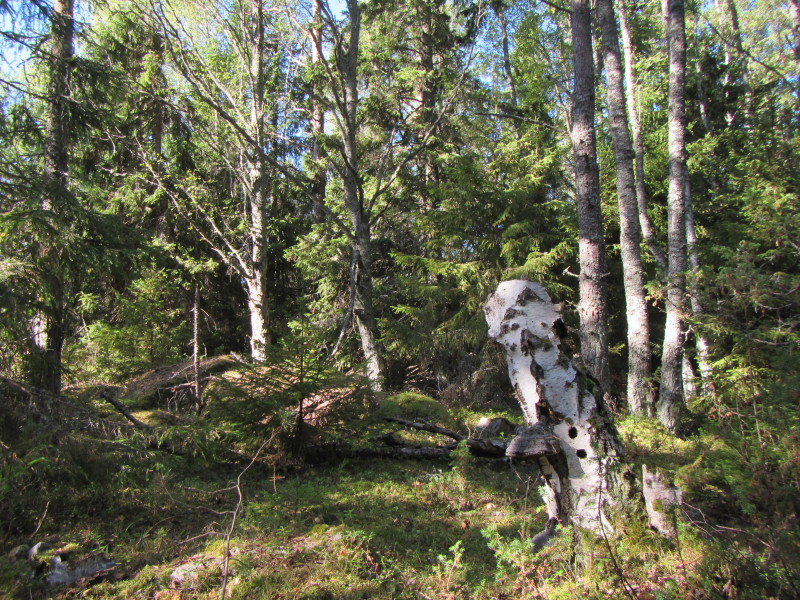
Habitat of *Didymium minus* and *Trichia favoginea* on the island of Boxö, in Saltvik municipality.

**Figure 5.**
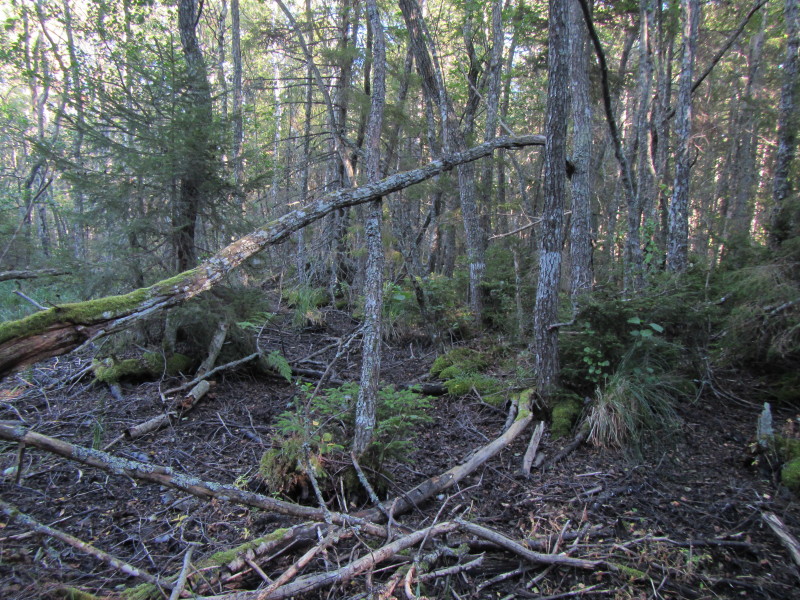
Habitat of *Hemitrichia clavata* on the island of Boxö ön, in Saltvik municipality.

The study sites were located on the islands north of the Åland main island (Fig. 1). All study sites were old-growth forest, but dominance of tree species and forest habitat types varied a lot. On Svartnö habitat was *Picea abies* dominated forest (Fig. 2) and *Pinus sylvestris* was the dominant tree species in the forests of Finbo (Fig. 3). The habitat on Boxö was mixed herb-rich forest Fig. 4﻿ and the habitat on Boxö ön was deciduous tree dominated forest with *Alnus* and *Salix* (Fig. 5). The island of Boxö ön has been part of nature reserve since 1988 (Kulves 2004).

**Data collection**

The material was collected in the Åland Islands 30.VIII.-6.IX.2014 by the authors Panu Kunttu and Sanna-Mari Kunttu. Altogether 145 myxomycete specimens were collected. The inventory was made by method of opportunistic sampling of species (Stokland and Sippola 2004). Interesting looking substrates, mainly dead wood, were selected for inventory based on their appearance and known habitat requirements of myxomycetes. Specimens were selected by sight and only fully-developed sporocarps were collected. Specimens were dried immediately after sampling and were preserved into the cartons. Identification of specimens was done microscopically two months after the sampling. The main goal of inventory was to contribute knowledge of myxomycete species occurring in the Åland Islands and gain information about ecology of infrequently collected or rare myxomycetes. Therefore specimens were collected selectively from different kind of forest habitat types.

Most of the specimens were identified by Elina Varis and Marja Härkönen helped with few specimens. Voucher specimens are deposited in the Herbarium of Turku University (TUR). Coordinates are given both in World Geodetic System (WGS84) and Finnish National Uniform Coordinate System (UCS), the latter is according to Heikinheimo and Raatikainen 1981. Nomenclature follows Härkönen and Varis 2012 and an online nomenclatural information system of Eumycetozoa (Lado 2014). Decay stage classification (1-5) of trunks is according to Renvall (1995), where stage 1 meaning fresh dead wood and 5 completely decayed.

## Taxon treatments

### Comatricha elegans

(Racib.) G. Lister

#### Materials

- **Type status:** Other material. **Location:** continent: Europe; country: Finland; stateProvince: Åland Islands; municipality: Eckerö; locality: Finbo, Brännsvikkärret; verbatimCoordinates: UCS 6716:3091; verbatimLatitude: 60.3499; verbatimLongitude: 19.5800; **Identification:** identifiedBy: Elina Varis; Marja Härkönen; dateIdentified: 2014-11; **Event:** eventDate: 2014-09-01; habitat: *Pinus sylvestris*, fallen trunk, diam. 17 cm, decay stage 3. Type of the trunk was a kelo tree, i.e. hard and grey, decorticated surface of the trunk.; **Record Level:** collectionID: Panu Kunttu 8401; institutionCode: TUR

#### Description

As presented in Härkönen and Varis 2012

### Cribraria intricata

Schrad.

#### Materials

- **Type status:** Other material. **Location:** continent: Europe; country: Finland; stateProvince: Åland Islands; municipality: Eckerö; locality: Svartnö; verbatimCoordinates: UCS 6708:3093; verbatimLatitude: 60.2833; verbatimLongitude: 19.6358; **Identification:** identifiedBy: Elina Varis; dateIdentified: 2014-11; **Event:** eventDate: 2014-09-02; habitat: *Pinus sylvestris*, fallen broken trunk, diam. 29 cm, decay stage 5. Type of the trunk was a kelo tree, i.e. hard and grey, decorticated surface of the trunk.; **Record Level:** collectionID: Panu Kunttu 8461; institutionCode: TUR

#### Description

As presented in Härkönen and Varis 2012.

### Didymium minus

Lister (Morgan)

#### Materials

- **Type status:** Other material. **Location:** continent: Europe; country: Finland; stateProvince: Åland Islands; locality: Saltvik; verbatimLocality: Boxö, Långnäs; verbatimCoordinates: UCS 6719:3121; verbatimLatitude: 60.4044; verbatimLongitude: 20.1147; **Identification:** identifiedBy: Elina Varis; dateIdentified: 2014-11; **Event:** eventDate: 2014-09-04; habitat: Hay in herb-rich forest; **Record Level:** collectionID: Panu Kunttu 8527; institutionCode: TUR

#### Description

As presented in Härkönen and Varis 2012

### Hemitrichia clavata

Pers. Rostaf.

#### Materials

- **Type status:** Other material. **Location:** continent: Europe; country: Finland; stateProvince: Åland Islands; municipality: Saltvik; locality: Boxö ön, Mararna; verbatimCoordinates: UCS 6716:3121; verbatimLatitude: 60.3764; verbatimLongitude: 20.1212; **Identification:** identifiedBy: Elina Varis; Marja Härkönen; dateIdentified: 2014-11; **Event:** eventDate: 2014-09-05; habitat: *Alnus glutinosa*, fallen trunk, diam. 14 cm, decay stage 3; **Record Level:** collectionID: Panu Kunttu 8579; institutionCode: TUR
- **Type status:** Other material. **Location:** continent: Europe; country: Finland; stateProvince: Åland Islands; municipality: Saltvik; locality: Boxö ön, Mararna; verbatimCoordinates: UCS 6716:3121; verbatimLatitude: 60.3764; verbatimLongitude: 20.1212; **Identification:** identifiedBy: Elina Varis; dateIdentified: 2014-11; **Event:** eventDate: 2014-09-05; habitat: *Alnus glutinosa*, fallen branch, diam. 3 cm, decay stage 3; **Record Level:** collectionID: Panu Kunttu 8582; institutionCode: TUR

#### Description

As presented in Härkönen and Varis 2012

### Licea variabilis

Schrad.

#### Materials

- **Type status:** Other material. **Location:** continent: Europe; country: Finland; stateProvince: Åland Islands; municipality: Eckerö; locality: Finbo, Brännsvikkärret; verbatimCoordinates: UCS 6716:3091; verbatimLatitude: 60.3499; verbatimLongitude: 19.5800; **Identification:** identifiedBy: Elina Varis; dateIdentified: 2014-11; **Event:** eventDate: 2014-09-01; habitat: on *Pinus sylvestris*, fallen branch, diam. 5 cm, decay stage 2; **Record Level:** collectionID: Panu Kunttu 8436; institutionCode: TUR

#### Description

As presented in Härkönen and Varis 2012.

### Trichia favoginea

(Batsch) Pers.

#### Materials

- **Type status:** Other material. **Location:** continent: Europe; country: Finland; stateProvince: Åland Islands; municipality: Saltvik; locality: Boxö, Långnäs; verbatimCoordinates: UCS 6718:3121; verbatimLatitude: 60.4044; verbatimLongitude: 20.1147; **Identification:** identifiedBy: Elina Varis; dateIdentified: 2014-11; **Event:** eventDate: 2014-09-04; habitat: *Betula* sp., fallen trunk, diam. 23 cm, decay stage 4; **Record Level:** collectionID: Panu Kunttu 8519; institutionCode: TUR

#### Description

As presented in Härkönen and Varis 2012.

## Discussion

Six myxomycete species new to the Åland Islands were found. Four of them - *Didymium minus*, *Hemitrichia clavata*, *Licea variabilis*, *Trichia favoginea* - were expected to be found from the Åland Islands, because these species are fairly common in Finland and species have been found from many biogeographical provinces around Finland.

The collection of *Cribraria intricata* was the third record in Finland. Earlier records are from Lohja (Regio aboënsis) and Tampere (Tavastia australis), in Southern Finland. Sporocarps of this species have been found from woody debris (Härkönen and Varis 2012). *C. intricata* is a cosmopolitan species and it is common in tropics (Härkönen and Varis 2012). It has been found for example from United States, Ecuador, Turkey, Taiwan, Mexico, many African countries and New Caledonia (Wann and Muenscher 1922, Schnittler et al. 2002, Ocak and Hasenekoğlu 2005, Ya-Fen et al. 2005, Moreno et al. 2001, Ndiritu et al. 2009, Kylin et al. 2013). Substrates have been dead wood and bark of living trees.

Probably due to its tiny sporocarp, *Comatricha elegans* is rarely found species in Finland; it has been found earlier only from two biogeographical province in Finland: Regio aboënsis and Nylandia in Southern Finland.

The Åland Islands are a biogeographically inadequately known part of Finland because of its somewhat remote location from continental part of Finland. In general, occurrence of myxomycetes is poorly known also in many other parts of Finland. The accumulation of knowledge of myxomycetes’ distribution is slow because there are only few researchers in Finland who are specialized on myxomycetes.

There are now 55 species of myxomycetes found in the Åland Islands. Comparing this number of species to other biogeographical provinces in Southern Finland, it is obvious that dozens of species can still be found there. For example, many common species in genera *Arcyria*, *Didymium* and *Physarum* are still undiscovered in the Åland Islands (Härkönen and Varis 2012). The species list of adjacent biogeographical province Regio aboënsis comprises 124 myxomycete species (Härkönen and Varis 2012). The main island of Åland is situated much closer to Sweden mainland than continental parts of Finland, so myxomycete species found in Sweden is also possibly to find from the Åland Islands. Swedish list of species contains 220 myxomycete species (Eliasson and Gilert 2007, Eliasson and Adamonyte 2009).

## Supplementary Material

XML Treatment for Comatricha elegans

XML Treatment for Cribraria intricata

XML Treatment for Didymium minus

XML Treatment for Hemitrichia clavata

XML Treatment for Licea variabilis

XML Treatment for Trichia favoginea
